# New software

**DOI:** 10.1155/S1463924601000025

**Published:** 2001


Journal of Automated Methods & Management in Chemistry, Vol. 23, No. 1 (January–February 2001) pp. 39

### New software

### Spellex announces world's first biotechnology and pharmaceutical spell checking software

Spellex Development announces the release of *Spellex Biotech* and *Spellex Pharmaceutical*, two new speciality software programs for personal computer users in the biotechnology and pharmaceutical sectors. The *Spellex Bio/Rx* software bundle integrates with *Microsoft Word*, *WordPerfect* or *Lotus* and helps users to check the spelling of over 200 000 biotechnology and pharmaceutical terms without leaving their word processing program.

*Benefits*

*Spellex Bio/Rx* spell checking software bundle helps PC users who work with bioscience and pharmaceutical terminology to create more professional looking documents quickly and accurately. With both *Spellex Bio/Rx* software installed, *Microsoft*, *WordPerfect* and *Lotus* spell checkers will provide correct spelling choices for incorrectly spelled biotechnology and pharmaceutical terms. The spell checkers also permit users to look up the spelling of biotechnology and pharmaceutical words of which they are unsure by phonetic or wildcard searching without leaving their document. Users save time, increase accuracy and improve productivity.

*Biotechnology*

*Spellex Biotech* provides a comprehensive spell checking solution for biotechnology field covering dozens of bioscience specialities including molecular biology, biomedicine, bioinformatics, general biology, general chemistry, organic chemistry, biochemistry, biophysics, botany, microbiology, cell biology, molecular genetics and the life sciences. *Spellex Biotech* spell checks biochemical nomenclature and alternative names for biochemical compounds and includes hundreds of pertinent abbreviations, acronyms, eponyms and organizations useful to biochemical and molecular biologists.

*Pharmaceuticals*

*Spellex Pharmaceutical* includes OTC and prescription pharmaceuticals including generic and trade drug names, orphan drugs, investigational drugs, chemotherapy drugs, drug protocols, Latin and Greek terms, pharmaceutical manufacturers, drug distributors and more. *Spellex Pharmaceutical* also covers pharmacologic–therapeutic classifications containing anesthetics, antihistamine drugs, anti-infective agents, antineoplastic agents, auto-nomic drugs, cardiovascular drugs, central nervous system agents, diagnosic agents, enzymes, gastrointestinal drugs, hormones, oxytocics, serums, vaccines, vitamins and more.

*Spell-X-Bio*™

*Spell-X-Bio*™ is a software subscription service that keeps subscribers' programs up to date with the latest words in biotechnology and pharmacology. Every three months users can download the latest *Spell-X-Bio*™ releases from the Spellex web site enabling them to keep their spellers current with the ever-changing language of the bioscience and pharmaceutical industries.

*For information and pricing for the full line of Spellex spelling dictionaries for Microsoft, WordPerfect and Lotus, visit http://www.spellex.com or contact Spellex Development at custsvce@-spellex.com or 800-442-WORD(9673)*

DOI: 10.1080/14639240110043831

